# The Effect of Risk Accumulation on Childhood Stunting: A Matched Case-Control Study in China

**DOI:** 10.3389/fped.2022.816870

**Published:** 2022-05-31

**Authors:** Xiao Tang, Yanxiang Zhao, Qigui Liu, Dongmei Hu, Guorong Li, Jin Sun, Guirong Song

**Affiliations:** ^1^Department of Health Statistics, School of Public Health, Dalian Medical University, Dalian, China; ^2^Department of Mathematics, George Washington University, Washington, DC, United States; ^3^Child Health Care Clinic, Dalian Women and Children's Medical Group, Dalian, China

**Keywords:** childhood stunting, case-control study, risk accumulation, decision tree, risk factors

## Abstract

**Background:**

Childhood stunting is still a public health issue in developing countries. However, the traditional risk factors in underdeveloped areas are not suitable for developed areas. Moreover, childhood stunting is influenced by several aspects, including genetic factors, perinatal conditions, maternal conditions, and feeding practices, but researchers have not yet clearly determined which aspect of risk accumulation exerts the strongest effect on stunting. A matched case-control study was performed to assess the effect of different aspects of risk accumulation on childhood stunting.

**Methods:**

In total, 173 non-stunted children aged under 7 years were matched in our study from June 2015 to August 2015. The children's heights and weights were measured, and a self-administered questionnaire was used to collect information from the children and their parents. The risk factors were assigned to the following five aspects: genetic factors, family socioeconomic status, perinatal conditions, maternal conditions, and feeding practices. The risk accumulation (cumulative risk score) in each aspect was defined as the total number of risk factors that occurred in a certain aspect. A conditional logistic regression model was used to assess the effect of risk accumulation in different aspects on stunting, and a decision-tree model was used to predict the children's stunting based on the cumulative risk scores.

**Results:**

Risk accumulation in perinatal conditions, genetic factors, maternal conditions, and feeding practices was significant in the conditional logistic regression model (*P* < 0.05). Perinatal conditions showed the strongest association with stunting in both the regression analysis and the decision-tree model. The risk of stunting increased by 1.199 times if the cumulative risk score for perinatal conditions increased by one, and the probability of stunting was 75.8% if the cumulative risk score for perinatal conditions was ≥1.

**Conclusion:**

Risk accumulation in perinatal conditions, genetic factors, maternal conditions, and feeding practices substantially increased the probability of stunting in childhood. Perinatal conditions were the main aspect associated with stunting. Prevention and intervention measures should be adopted to avoid risk accumulation in stunting.

## Introduction

Stunting is defined as a height-for-age index less than two standard deviations below the median World Health Organization (WHO) Child Growth Standard and is an acknowledged indicator representing the child health inequalities (1). Stunting is the result of chronic or recurrent undernutrition and leads to increased morbidity and mortality rates. Stunting is associated with future negative consequences, such as poor cognitive development, impaired physical growth, lower economic productivity, and a greater risk of developing overweight, obesity, and non-communicable diseases in adulthood (2–5). Furthermore, stunting even has an intergenerational effect, resulting in growth retardation in offspring (6, 7).

Child growth retardation is still an important problem in underdeveloped and developing countries. In 2012, the WHO set global targets for maternal and child nutrition, the first of which is to reduce the number of stunted children aged under 5 years by 40% by 2025 (8). Although the national prevalence of stunting in China has significantly decreased over the past two decades, disparities remain among age, provincial, and ethnic groups due to unequal economic levels and diverse cultures (9). Stunting remains a public health concern in China, even in urban areas.

The determinants of stunting are multidimensional and have obvious regional characteristics. Understanding the roles of risk factors in childhood stunting is very important for proposing targeted strategies to alleviate this problem. Previous studies investigated the determinants of stunting to develop prevention and control strategies (7, 10–13). However, these studies were mostly conducted in underdeveloped areas, and thus, the risk factors of stunting in these areas included poor wealth status, poor sanitary practices, and inadequate healthcare utilization, such as unprotected sources of water, delivery at home, unavailability of latrines, number of siblings, non-exclusive breastfeeding, and insufficient food intake (10–13). In urban areas of China, the publicsanitary situation and infrastructure have substantially improved, most families are able to provide adequate food to pregnant women and children, and pregnant women are required to follow standardized healthcare procedures (14, 15). Furthermore, China has insisted on the one-child policy for more than three decades. Most families have only one child, and each family provides almost the best conditions for their children. Therefore, some traditional risk factors in underdeveloped countries are inapplicable to developed regions. Researchers have not yet clearly identified the key factors responsible for childhood stunting in urban areas of China.

Furthermore, previous studies have shown that the determinants of childhood stunting involve several aspects, such as genetics, socioeconomic status, healthcare, nutrition, and environmental situation, each of which still includes many factors (7, 10–13). Most research focused on paramount factors (independent factors) among a large number of factors or only explored a certain aspect influencing stunting (7, 10–13). However, risk accumulation increases the probability of stunting, although the specific aspect of risk accumulation that exerts the strongest effect on stunting is unclear. Therefore, studying risk accumulation in various aspects could be critical for preventing stunting in children, revealing the aspect of risk accumulation that exerts the strongest effect, and guiding the development of prevention/intervention/alleviation strategies for stunting.

Dalian is a modern coastal city in northeast China whose economy and environment significantly differ from those of underdeveloped areas. Although the prevalence of stunting in Dalian has shown a steady decreasing trend and is lower than the national average beginning in 2,000, stunting is still a public concern in Dalian (16, 17). Our aim is to identify the determinants of stunting in children aged under 7 years in Dalian and assess the effects of risk accumulation in different dimensions on stunting. Moreover, we established a classification scheme using a decision-tree model to predict the occurrence of stunting based on the cumulative score for each dimension.

## Methods

### Selection of Subjects and Research Design

In our city, pregnant women are asked to follow standardized prenatal checkup procedures and build medical records from early pregnancy, which is approximately 4 gestational weeks, and preschool children are required to undergo routine health checkups in community health service stations (CHSS). A 1:1 matched case-control study of children aged under 7 years in Dalian was conducted. First, 12 CHSS were selected from three districts and four counties in Dalian City, China using a stratified sampling technique. Districts and counties were defined as strata, and one or two CHSS were randomly selected from each district or county based on the medical service capacity for children at the CHSS.

Second, children with stunting were recruited from June 2015 to August 2015 from 12 CHSS, and the stunted children were defined as having a height more than two standard deviations below the median child growth standard by sex and age according to the growth and development standards for children under 7 years in China (18). Meanwhile, normally developing children undergoing health checkups in the same CHSS were recruited as the control group and matched with the cases. Each pair of cases and controls was matched according to sex, administrative district, and age. Specifically, when the case was younger than 1 year, the age difference between the case and the control was controlled to <1 week, and when the case was older than 1 year, the age difference was controlled to <1 month.

The inclusion criteria were children with permanent urban or rural household registration in Dalian and a complete physical examination, and their parents provided informed consent and volunteered to participate in the study. Children who had endemic diseases, tuberculosis, chronic nephritis, delayed hepatitis, asthma, chronic bronchitis, heart disease, nervous system diseases, endocrine diseases, rickets (above medium degree), or other deformities or lived locally for less than two-thirds of their age were excluded from the study.

We estimated the appropriate sample size based on the results of similar studies in which the odds ratios (ORs) of different factors ranged from ~1.5 to 5. Setting the significance level to 0.05, OR = 2, and statistical power = 80%, the final estimated sample size was 340 subjects, including 170 cases and 170 controls. In total, 175 matched pairs were recruited for our study.

The study was approved by the ethics committee of Dalian Medical University. Written informed consent was provided by all participants. The research was conducted in accordance with the Declaration of Helsinki.

### Anthropometric Measurements

Weight was measured with the children wearing minimal clothing and bare feet. Weight was recorded in kilograms. For the children aged under 2 years, a length board with a sliding headpiece was used to measure their height. These children lay in the supine position along the middle of the board, and trained nurses moved the sliding headpiece to touch their heads. Children aged 2–6 years stood against the stadiometer on the board, and trained nurses measured their heights. Newborns were placed in the supine position with flattened knee joints and straightened lower limbs by nurses. Then, the distance from the top of the head to the heel, measured with a soft ruler, was recorded as the length at birth. Height was recorded in centimeters.

### Questionnaire Collection and Independent Variables

#### Development of the Questionnaire

A questionnaire was constructed and used to collect the information about the children and parents. In total, 13 specialists in child healthcare, high-risk infant and premature infant subspecialties, child nutrition, child growth and development subspecialties, and epidemiology participated in the development of the questionnaire. First, Director Sun wrote a draft based on years of work experience and related references (7, 19–21), and then, 13 specialists discussed the items in six meetings and repeatedly modified the questionnaire. Finally, 27 items in the questionnaire were generally based on the traditional determinants of stunting and some suitable factors in urban areas and were divided into five dimensions. Before the formal investigation, the practicality and universality of the questionnaire were verified by a pilot survey conducted in several CHSS. Some parents with a low education level were able to fully understand the statements in the questionnaire with the help of the investigators.

#### Reliability and Validity of the Questionnaire

Item reduction was completed by the following multiple methods: (1) exploratory factor analysis: the items had a factor loading of 0.4 or lower; (2) Cronbach's alpha coefficient: an item caused the coefficient to increase by 0.1 or greater when it was deleted; and (3) discrimination analysis: the difference in the items between the case and control groups was not statistically significant at the 0.05 level. The items that met at least two of the above requirements were deleted. Hence, the following six items were deleted: “annual incomes,” “pregnancy complications,” “birth asphyxia or admission to the neonatal intensive care unit (NICU),” “placenta previa or placental shape aberrations,” “bigeminal pregnancy or multiple pregnancies,” and “timing of complementary feeding.”

The Cronbach's alpha coefficient of the final questionnaire with 21 items was 0.73. Construct validity was examined by performing a confirmatory factor analysis (CFA). The comparative fit index (CFI) is a comparative goodness-of-fit index. The analysis of the questionnaire produced a CFI value of 0.91, an X^2^/df of 1.83, and a root mean square of approximate error (RMSEA) of 0.05. In summary, the fitting effect of the questionnaire was very good.

#### Questionnaire Collection

When parents took their children to the CHSS for a physical examination, the medical staff, who had completed unified training, asked the parents the questions and completed the questionnaire. The responses to some items related to medical history, including perinatal conditions (intrauterine growth retardation, premature birth, birth length, birth weight, and vernix/meconium-stained amniotic fluid) and maternal conditions (weight gain and disease during pregnancy), were confirmed by the staff after checking the medical records. Two matched pairs were excluded because of incomplete questionnaires, resulting in 173 pairs for the analysis.

#### Independent Variables

The final questionnaire included the following five dimensions: genetic factors (four items), maternal conditions (five items), family socioeconomic status (three items), perinatal conditions (four items), and feeding practices (five items).

##### Genetic Factors

The genetic factors included parental height and childbearing age. A mother and father with a height less than the median minus one standard deviation were classified as short (short = 1, normal = 0). A mother and father with a childbearing age more than 35 years were identified as older (older = 1, younger = 0). The sum of the scores for each item (four items, range: 0–4 points) was defined as the cumulative risk scores for the genetic factors, and higher scores indicated greater risk accumulation in the genetic aspect.

##### Family Socioeconomic Status

The family socioeconomic status included parental education and the housing situation. Paternal education = 1 if the father had a high school degree or less, while paternal education = 0 if the father had a college degree or above. The variable of maternal education was defined the same as paternal education. Housing situation = 1 if the room was dark and dank, and housing situation = 0 if the room was bright. The sum of the scores for each item (three items, range: 0–3 points) was defined as the cumulative risk score for family socioeconomic status, and higher scores indicated greater risk accumulation in socioeconomic status.

##### Maternal Conditions

The maternal conditions included the following five items: (1) self-perceived nutrition situation during pregnancy (0 = good, 1 = not good); (2) perceived pickiness during pregnancy (1 = yes, 0 = no); (3) weight gain during pregnancy >9 kg (1 = no, 0 = yes); (4) diagnosed with a nutritional disease during pregnancy (1 = yes, 0 = no); and (5) diagnosed with vernix/meconium-stained amniotic fluid (1 = yes, 0 = no). Maternal weight gain during pregnancy was defined as the difference between the weight before delivery and that in early pregnancy, which was approximately 4 gestational weeks. The sum of the scores for each item (five items, range: 0–5 points) was calculated as the cumulative risk score of maternal conditions, and higher scores indicated greater risk accumulation in maternal conditions.

##### Perinatal Conditions

The following four characteristics of perinatal conditions were extracted from the questionnaire and medical records: (1) intrauterine growth retardation if the estimated fetal weight (EFW) on ultrasound was less than the 10th percentile of the standard fetal weight in China for the gestational week (1 = yes, 0 = no) (22); (2) premature baby if the gestational age was <37 weeks (1 = yes, 0 = no); (3) low birth weight if the birth weight was <2.5 kg (1 = yes, 0 = no); and (4) low birth length if the birth length was less than the median minus one SD (1 = yes, 0 = no). The sum of the scores for each item (four items, range: 0–4 points) was defined as the cumulative risk score for perinatal conditions, and higher scores indicated greater risk accumulation in perinatal conditions.

##### Feeding Practices

The following five characteristics of feeding were extracted from the questionnaire: (1) breastfeeding (0 = yes, 1 = no); (2) children's appetite (0 = good, 1 = bad); (3) chasing children to feed (0 = no, 1 = yes); (4) not swallowing food for a long time during meals (0 = no, 1 = yes); and (5) susceptibility to diarrhea (0 = no, 1 = yes). In our study, breastfeeding was defined as breastfeeding for at least 6 months from birth and consuming more than 80% of the total amount of breast milk. If a baby was younger than 6 months, breastfeeding was defined as consuming more than 80% of the total amount of breast milk. Susceptibility to diarrhea was defined as a child having diarrhea at least twice a month. The sum of the scores for each item (five items, range: 0–5 points) was defined as the cumulative risk score for feeding practices, and higher scores indicated greater risk accumulation in feeding practices.

The total score was the sum of each cumulative risk score described above and represents the risk accumulation of child stunting. Questions regarding the fathers' drinking and smoking habits (no, or yes) were also asked.

### Statistical Analysis

The data were entered into Microsoft Excel 2007, and the statistical analyses were performed using SPSS 25.0. The discrete variables are reported as frequencies and percentages, and a chi-square test was used to compare the difference in the factors between the case group and the control group. The reliability of our questionnaire was estimated with the Cronbach's α coefficient, and a CFA was performed to assess the construct validity of the questionnaire using Amos 25.0. A multivariable conditional logistic regression analysis with a stepwise method was conducted to identify the independent factors responsible for stunting among 21 variables. Univariate and multivariate conditional logistic regression analyses were performed to explore the association between stunting and the risk accumulation of genetic factors, family socioeconomic status, maternal conditions, perinatal conditions, and feeding practices.

All participants were divided into three groups according to the tri-sectional quantiles of the total score. The reference group scored 0–3 points, the middle group scored 4–6 points, and the highest group scored more than 7 points. A conditional logistic regression analysis was used to compare the risk of stunting between the higher score groups and the reference group. Model 1 was adjusted for fathers' smoking habits, and Model 2 was adjusted for fathers' smoking and drinking habits.

Subsequently, the decision-tree model in R 4.0.3 was used to explore the important variables and predict the probability of stunting based on the cumulative risk scores for genetic factors, family socioeconomic status, maternal conditions, perinatal conditions, and feeding practices. In this study, the dataset was randomly divided into the following two subsets: a training dataset containing approximately 70% of the children (279 cases) and a testing dataset containing 30% of the children (67 cases). The model was first constructed based on the training dataset to build the decision tree. In the tree, the first variable (the root of the tree) was the most important aspect, and the variables split from the root in an orderly manner were the next important aspects in classifying the data. All variables in one path were considered predictors (if part), and the class label of the leaf node was an expected outcome (then part).

In the decision tree, a “stunted child” was defined as a positive event S, and a “non-stunted child” was defined as a negative event N. Then, the model was tested using the testing subset. Using the rules from the decision tree to assess the testing dataset, a confusion matrix showing true positives, true negatives, false positives, and false negatives of the testing dataset was obtained. We calculated the accuracy, sensitivity, and specificity to measure the performance of the model.

## Results

### Comparison of the General Characteristics of Stunted and Non-Stunted Children

The basic characteristics of the case group and the control group are displayed in Table 1. Almost all factors significantly differed between these two groups, except for the maternal childbearing age, paternal education, maternal education, and fathers' drinking and smoking habits. The stunted children were more likely to have a short mother, a short father, an older father at birth, a lower birth weight, a lower birth length, intrauterine growth retardation, worse appetite, and vulnerability to diarrhea. The mothers of the stunted children were more likely to have stained amniotic fluid, poor nutrition, nutritional disease, picky eating habits, and lower weight gain during pregnancy. The stunted children had a greater probability of living in families that had worse housing conditions, and their parents were more likely to chase the children to feed food and not to breastfeed.

**Table 1:** Differences in determinants associated with stunting between the case group and the control group.

**Variables**	**Case group**	**Control group**	**OR (95% CI)**	**χ^2^**	***P***
	***n* (%)**	***n* (%)**			
**Genetic factors**					
**Paternal bearing age**					
<35 years	102 (59.0)	129 (74.6)			
≥35 years	71 (41.0)	44 (25.4)	2.200 (1.369, 3.817)	9.495	0.002
**Maternal bearing age**					
<35 years	127 (73.4)	140 (80.9)			
≥35 years	46 (26.6)	33 (19.1)	1.810 (1.062, 3.038)	2.772	0.096
**Paternal height**					
Short	35 (20.2)	4 (2.3)	11.333 (3.481, 36.900)	27.771	<0.001
Not short	138 (79.8)	169 (97.7)			
**Maternal height**					
Short	47 (27.2)	3 (1.7)	23.000 (5.583, 94.747)	45.261	<0.001
Not short	126 (72.8)	170 (98.3)			
**Family socioeconomic status**
**Paternal education**					
High school or less	123 (71.1)	117 (67.6)	1.177 (0.745, 1.861)	0.490	0.484
College or above	50 (28.9)	56 (32.4)			
**Maternal education**					
High school or less	124 (71.7)	124 (71.7)	1.000 (0.626, 1.596)	0.000	1.000
College or above	49 (28.3)	49 (28.3)			
**Housing conditions**					
Small, dark and damp	20 (11.6)	1 (0.6)	20.000 (2.684, 149.022)	18.301	<0.001
Spacious, bright and airy	153 (88.4)	172 (99.4)			
**Maternal conditions during pregnancy**
**Maternal weight gain**					
<9 kg	51 (29.5)	16 (9.2)	4.102 (2.230, 7.545)	22.674	<0.001
≥9 kg	122 (70.5)	157 (90.8)			
**Stained amniotic fluid**					
Yes	11 (6.4)	2 (1.2)	5.806 (1.267, 26.594)	6.474	0.011
No	162 (93.6)	171 (98.8)			
**Maternal nutrition**					
Poor	84 (48.6)	32 (18.5)	4.159 (2.558, 6.761)	35.067	<0.001
Good	89 (51.4)	141 (81.5)			
**Nutritional disease**					
Yes	24 (13.9)	11 (6.4)	2.372 (1.123, 5.009)	5.372	0.02
No	149 (86.1)	162 (93.6)			
**Picky eating habits**					
Yes	42 (24.3)	15 (8.7)	3.377 (1.793, 6.362)	15.312	<0.001
No	131 (75.7)	158 (91.3)			
**Perinatal conditions**					
**Birth weight**
Low	41 (23.7)	11 (6.4)	4.574 (2.262, 9.249)	20.369	<0.001
Normal	132 (76.3)	162 (93.6)			
**Birth length**
Low	88 (50.9)	21 (12.1)	7.494 (4.345, 12.924)	60.124	<0.001
Normal	85 (49.1)	152 (87.9)			
**Intrauterine growth retardation**
Yes	14 (8.1)	5 (2.9)	2.958 (1.042, 8.403)	4.511	0.034
No	159 (91.9)	168 (97.1)			
**Premature infant**
Yes	30 (17.3)	8 (4.6)	4.327 (1.922, 9.740)	14.308	<0.001
No	143 (82.7)	165 (95.4)			
**Feeding practices**					
**Breastfeeding**
Yes	148 (85.5)	166 (96.0)			
No	25 (14.5)	7 (4.0)	4.006 (1.683, 9.532)	11.157	0.001
**Children's appetite**
Good	50 (28.9)	112 (64.7)			
Poor	123 (71.1)	61 (35.3)	4.517 (2.871, 7.106)	44.620	<0.001
**Chasing children to feed**
Yes	55 (31.8)	37 (21.4)	1.713 (1.056, 2.780)	4.797	0.029
No	118 (68.2)	136 (78.6)			
**Not swallowing food for a long time during meals**
Yes	26 (15.0)	11 (6.4)	2.276 (1.132, 4.577)	5.541	0.019
No	147 (85.0)	162 (93.6)			
**Susceptibility to diarrhea**					
Yes	26 (15.0)	11 (6.4)	2.605 (1.243, 5.457)	6.809	0.009
No	147 (85.0)	162 (93.6)			
**Other factors**					
**Fathers' drinking habit**					
Yes	32 (18.5)	31 (17.9)	1.071 (0.517, 2.220)	0.019	0.889
No	141 (81.5)	142 (82.1)			
**Fathers' smoking habit**					
Yes	64 (37.0)	68 (39.3)	0.886 (0.546, 1.436)	0.242	0.623
No	109 (63.0)	105 (60.7)			

*OR, odds ratio; CI, confidence interval*.

### The Effect of Risk Accumulation in Different Aspects on Stunting

The independent factors for stunting identified by the multivariable conditional logistic regression analysis are shown in Supplementary Table 1. The paternal childbearing age, maternal height, birth length, and children's appetite were independent factors for stunting. A paternal childbearing age ≥35 years, short mother, short birth length, and poor appetite increased the probability of stunting.

The associations between stunting and genetic factors, maternal conditions, family socioeconomic status, perinatal conditions, and feeding practices are shown in Table 2. The risk accumulation in each dimension was significant in the univariate models. However, in the multivariate model, only the risk accumulations in genetic factors, maternal conditions, perinatal conditions, and feeding practices were significant. Perinatal conditions showed the strongest association with stunting. The risk of stunting would increase by 1.199 times if the cumulative risk scores for perinatal conditions increased by one.

**Table 2:** The effect of the cumulative scores for different dimensions on childhood stunting by conditional logistic models.

**Variables**	**Univariate model**		**Multivariate model**	
	**OR (95% CI)**	***P***	**OR (95% CI)**	***P***
Genetic factors	2.506 (1.818, 3.455)	<0.001	1.609 (1.110, 2.333)	0.012
Family socioeconomic status	1.328 (1.044, 1.688)	0.021	0.961 (0.683, 1.352)	0.818
Maternal conditions	2.393 (1.762, 3.251)	<0.001	1.541 (1.055, 2.252)	0.025
Perinatal conditions	3.140 (2.065, 4.774)	<0.001	2.199 (1.434, 3.371)	<0.001
Feeding practices	1.980 (1.534, 2.556)	<0.001	1.421 (1.063, 1.899)	0.018

*OR, odds ratio; CI, confidence interval*.

*All models were adjusted for fathers' smoking and drinking habits*.

The risk of stunting among different total score groups is presented in Table 3. In Models 1 and 2, the risk of stunting increased by 3.242 and 3.236 times, respectively, in the group with cumulative risk scores between 4 and 6 points, and the risk of stunting increased by 13.885 and 13.844 times, respectively, in the group with the highest scores compared with the reference group (the lowest risk scores).

**Table 3:** The association between the total score and childhood stunting by conditional logistic models.

**Total score**	**Crude analyses**		**Model 1** ^†^		**Model 2** ^**‡**^	
	**OR (95% CI)**	***P***	**OR (95% CI)**	***P***	**OR (95% CI)**	***P***
0–3	–		–		–	
4–6	4.191 (2.110, 8.322)	<0.001	4.242 (2.119, 8.491)	<0.001	4.236 (2.115, 8.486)	<0.001
≥7	14.774 (6.743, 32.369)	<0.001	14.885 (6.771, 32.725)	<0.001	14.844 (6.749, 32.748)	<0.001

*OR, odds ratio; CI, confidence interval*.

*Model 1 was adjusted for fathers' smoking habits*.

*^‡^Model 2 was adjusted for fathers' smoking and drinking habits*.

### Rules for Predicting Stunting

The decision-tree model was evaluated using a confusion matrix on the testing dataset, and the results are displayed in Table 4. This model had an accuracy of 82.1%; 55 of 67 individuals were classified correctly, whereas 17.9% (12 of 67 individuals) were classified incorrectly. Of the 29 participants without stunting in the testing dataset, 24 were classified correctly using the decision tree with a specificity of 82.8%. Of the 38 individuals with stunting in the testing dataset, the decision tree correctly classified 31 individuals, with a sensitivity of 81.6%.

**Table 4:** Confusion matrix of the test dataset.

**Actual outcome**	**Predicted outcome**	
	**Children without stunting**	**Children with stunting**
Children without stunting	24	5
Children with stunting	7	31
Accuracy	82.1%	
Sensitivity	81.6%	
Specificity	82.8%	

**Figure 1:**
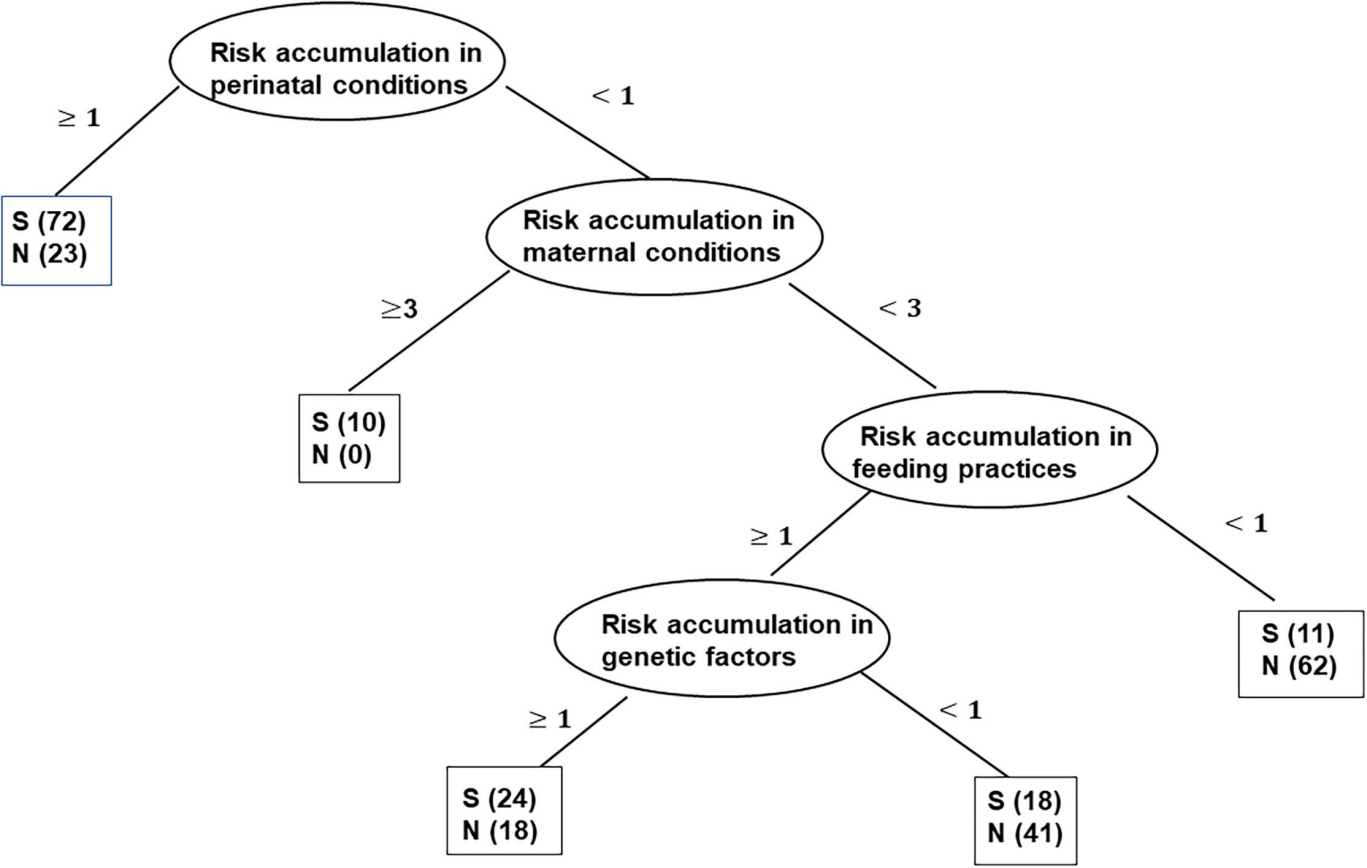
Graphical representation of the decision-tree model of the dataset. Sample sizes are shown in the brackets of each node. S, stunted child; N, non-stunted child.

As shown in Figure 1, the decision tree was constructed based on five input variables (cumulative risk scores for five dimensions) of the participants, and four remained in the model. The final decision tree contained six leaves and five layers. The IF-THEN rules created by the model are fully described in Table 5. The most important variable (root) in the tree was perinatal conditions. The probability of stunting was 75.8% if the cumulative risk score for perinatal conditions was ≥1. The second most important variable was maternal conditions. Children without a perinatal risk but with a cumulative risk score for maternal conditions ≥3 were identified as stunted. The third and fourth most important variables were feeding practices and genetic factors, respectively. Children who did not have perinatal risk and had a cumulative risk score for maternal conditions <3 and a cumulative risk score for feeding practices <1 had an 84.9% probability of not being stunted.

**Table 5:** Rules extracted through the decision-tree model.

Rule 1. If the cumulative risk scores for perinatal conditions ≥1, then children with stunting (72/95, 75.8%).
Rule 2. If the cumulative risk scores for perinatal conditions <1 and cumulative risk score for maternal conditions≥3, then children with stunting (10/10, 100%).
Rule 3. If the cumulative risk score for perinatal conditions <1, the score for maternal conditions <3, and the score for feeding practices <1, then children without stunting (62/73, 84.9%).
Rule 4. If the cumulative risk score for perinatal conditions <1, the score for maternal conditions <3, the scores for feeding practices ≥1 *and* the scores for genetic factors ≥1, then children with stunting (24/42, 57.1%).
Rule 5. If the cumulative risk scores for perinatal conditions <1, the score for maternal conditions <3, the score for feeding practices ≥1 *and* the score for genetic factors <1, then children without stunting (41/59, 69.5%).

## Discussion

In our study, an old father, a short mother, a short birth length, and a poor appetite were identified as independent factors for stunting using conventional analytical methods; however, half of these factors were genetic factors, which are relatively difficult to change. In addition, family socioeconomic status was not a critical aspect influencing stunting in developed cities. Moreover, our findings showed that risk accumulation in perinatal conditions, maternal conditions, and feeding practices played more important roles in stunting than genetic factors and family socioeconomic status, which could benefit the development of prevention and intervention strategies.

In this study, perinatal conditions played the most important role in childhood stunting. Evidence suggests that intrauterine growth retardation, low birth weight, low birth length, and preterm birth are strongly associated with stunting. A study conducted in Indonesia using national data showed that low birth weight was the major determinant of stunting (23). A similar result was observed in a survey in Ethiopia (11). A meta-analysis of 19 studies showed that children aged under 5 years with a low birth weight had a threefold higher risk of stunting (17). Intrauterine growth retardation has also been identified as a risk factor for stunting, wasting, and micronutrient deficiencies in children (24, 25). It is estimated that approximately one-fifth of stunted children have linkages to the fetal period as being born small for gestational age (SGA) (25). No effective therapies are available to reverse intrauterine growth restriction; hence, the focus should be on preventive strategies (26). In mothers with eating disorders and unusual or fad diets, it has been suggested that they have a higher probability of having babies with intrauterine growth restriction and low birth weight in developed countries, while in developing countries, caloric supplementation during pregnancy exerts the largest short-term effect on intrauterine growth (27). In addition, micronutrient supplementation, including iron, vitamin A, calcium, and multiple micronutrients, has shown a consistent association with a decrease in intrauterine growth restriction and increased birth weight (28–30).

In the current study, maternal conditions, including maternal weight gain, nutritional status, diet habits, and amniotic fluid contamination during pregnancy, were associated with stunting. In the decision-tree model, even if no perinatal risk factors were present, the probability of stunting was still high when more than three risk factors for maternal conditions were present. Maternal undernutrition during pregnancy is the traditional determinant of stunting (11, 31). However, in developed regions, maternal undernutrition might be due to a lack of pregnancy knowledge, morning sickness, and an unbalanced diet (28, 31, 32). In addition, stunted children have a higher proportion of exposure to amniotic fluid contamination than healthy children. Amniotic fluid contamination may lead to adverse health consequences in newborns, such as neonatal pneumonia and hypoxic-ischaemic encephalopathy, which might also affect their growth and development (33, 34).

Many previous studies have found that stunting is closely related to feeding practices, such as breastfeeding and complementary feeding, which is partially consistent with our results (35–39). A survey in 12 central and western provinces of China found that children who were primarily cared for by fathers received more inappropriate breastfeeding and had a higher risk of stunting than those cared for by mothers (35). A cross-sectional study based on 42 rural counties in seven western provinces of China showed that 44.5% of children aged 6–23 months failed to reach the minimum dietary diversity criteria defined by the WHO (36). Many studies have shown that the early initiation of breastfeeding and exclusive breastfeeding are protective factors against stunting, which was also supported in this study (37–39). In addition, we focused on the effect of eating behaviors and children's appetites on stunting. In this study, a poor appetite, not swallowing food, and chasing children to feed were risk factors for stunting. Children with poor appetite and unhealthy dietary habits might not consume balanced and diverse foods, resulting in malnutrition and stunting. Feeding knowledge should be promoted and provided (36–40). A cluster-randomized controlled trial in Laishui, China, showed that an educational intervention targeting caregivers' feeding practices might improve caregivers' knowledge and practices related to complementary feeding, thereby decreasing the probability of stunting in children aged under 3 years (40).

Our analysis revealed that parental height and childbearing age were associated with offspring stunting. A child born to a shorter mother or a shorter father has a greater probability of being stunted. Comparable findings were reported in other previous studies. Some studies have shown that the maternal height or body mass index (BMI) is inversely correlated with children's stunting (6, 11), which might be explained by the biological plausibility that shorter mothers may have a worse nutritional status and poorer health situation and fail to deliver adequate nutrition to the fetus during pregnancy (41). Others claimed that the stature of children is influenced by both maternal and paternal heights (42), which is consistent with our results. These findings signify the intergenerational effect of malnutrition. In this study, children of older mothers (aged more than 35 years) were more likely to be stunted than those of younger mothers (aged <35 years). This result is similar to the findings in Ghana, where stunting was associated with advanced maternal age (30–44 years) (43). However, this finding contrasts with the result in Tanzania, where younger mothers (aged <25 years) were more likely to have stunted children than older mothers (aged >25 years) (7). The reason for the discrepancy may lie in the fact that young mothers might have limited access to socioeconomic resources to meet the nutritional needs of their children in underdeveloped areas.

Our findings suggest that the effect of risk accumulation in different dimensions should not be ignored. Monitoring nutrition during pregnancy plays an important role in preventing intrauterine growth retardation, premature birth, low birth weight, and low birth height, which significantly influence children's height development. Education concerning feeding practices should still be advocated to decrease the probability of stunting, even if accumulated risks in maternal conditions and perinatal conditions are low.

This study has some limitations. First, due to the cross-sectional design, this study is limited in establishing a causal relationship between the observed risk factors and stunting. Future research should aim to investigate this possibility in a prospective study. Second, each item was assigned the same weight to calculate the scores for the different dimensions, which might not be true in practice. Third, the questionnaire used in this study was designed for subjects in developed areas; thus, the dimensions of the risk factors may be limited and not generalizable to other populations. Moreover, some non-traditional aspects, such as maternal psychosocial factors, were not included in the questionnaire, which needs further exploration. Some information concerning perinatal and maternal conditions, such as admission to the NICU, birth asphyxia, and maternal complications, may affect stunting, but we were unable to include these variables in our analysis due to their rare occurrence. Complementary feed is also a factor related to stunting, but our study lacked detailed information concerning complementary feeds, including quantity and type. Further investigations based on a large sample are needed to explore the relationship between these factors and stunting. Fourth, recall bias is a common disadvantage in case-control studies. In our study, some information was obtained from medical records, and some items were defined clearly and quantitatively. The medical staff, who had undergone unified training, asked the parents the questions, explained the items, and completed the questionnaire. All these measures helped control recall bias. Fifth, the factors within the same dimension were not completely independent, which may have biased the risk accumulation. Our study is the first to explore the association between risk accumulation and stunting, based on which better questionnaires should be constructed in future studies.

## Conclusion

Risk accumulation in perinatal conditions, genetic factors, maternal conditions, and feeding practices increased the probability of stunting in childhood. Perinatal conditions were the main factor associated with stunting. Prevention and intervention measures should be adopted to avoid risk accumulation in stunting.

## Data Availability Statement

The raw data supporting the conclusions of this article will be made available by the authors, without undue reservation.

## Ethics Statement

The studies involving human participants were reviewed and approved by Ethics Committee of Dalian Medical University. Written informed consent to participate in this study was provided by the participants' legal guardian/next of kin.

## Author Contributions

XT, YZ, and GS designed the study. QL, DH, and GL collected the data. All authors contributed to data analysis, drafting or revising the article, have agreed on the journal to which the article will be submitted, gave final approval of the version to be published, and agree to be accountable for all aspects of the work.

## Conflict of Interest

The authors declare that the research was conducted in the absence of any commercial or financial relationships that could be construed as a potential conflict of interest.

## Publisher's Note

All claims expressed in this article are solely those of the authors and do not necessarily represent those of their affiliated organizations, or those of the publisher, the editors and the reviewers. Any product that may be evaluated in this article, or claim that may be made by its manufacturer, is not guaranteed or endorsed by the publisher.

## References

[1] World Health Organization (WHO). WHO Child Growth Standards: Methods and Development. Geneva: Department of Nutrition for Health and Development, World Health Organization (2007).

[2] Hoddinott J Alderman H Behrman JR Haddad L Horton S. The economic rationale for investing in stunting reduction. Mater Child Nutr. (2013) 9(Suppl. 2):69–82. 10.1111/mcn.12080 24074319 PMC6860695

[3] Dewey KG Begum K. Long-term consequences of stunting in early life. Matern Child Nutr. (2011) 7(Suppl. 3):5–18. 10.1111/j.1740-8709.2011.00349.x 21929633 PMC6860846

[4] Victora CG Adair L Fall C Hallal PC Martorell R Richter L. Maternal and child undernutrition: consequences for adult health and human capital. Lancet. (2008) 371:340–57. 10.1016/S0140-6736(07)61692-4 18206223 PMC2258311

[5] Stein AD Thompson AM Waters A. Childhood growth and chronic disease: evidence from countries undergoing the nutrition transition. Matern Child Nutr. (2005) 1:177–84. 10.1111/j.1740-8709.2005.00021.x 16881898 PMC6860951

[6] Das S Alam MA Mahfuz M Arifeen SE Ahmed T. Relative contributions of the correlates of stunting in explaining the mean length-for-age z-score difference between 24-month-old stunted and non-stunted children living in a slum of Dhaka, Bangladesh: results from a decomposition analysis. BMJ Open. (2019) 9:e025439. 31366637 10.1136/bmjopen-2018-025439 PMC6678062

[7] Semali IA Tengia-Kessy A Mmbaga EJ Leyna G. Prevalence and determinants of stunting in under-five children in central Tanzania: remaining threats to achieving Millennium Development Goal 4. BMC Public Health. (2015) 15:1153. 10.1186/s12889-015-2507-6 26590803 PMC4654796

[8] Luo RF Shi Y Zhou H Yue A Zhang LX Sylvia S. Micronutrient deficiencies and developmental delays among infants: evidence from a cross-sectional survey in rural China. BMJ Open. (2015) 5:e008400. 10.1136/bmjopen-2015-008400 26438137 PMC4611485

[9] Zong XN Li H. Physical growth of children and adolescents in China over the past 35 years. Bull World Health Organ. (2014) 92:555–64. 10.2471/BLT.13.126243 25177070 PMC4147404

[10] Joe W Rajpal S Kim R Laxmaiah A Harikumar R Arlappa N. Association between anthropometric-based and food-based nutritional failure among children in India, 2015. Matern Child Nutr. (2019) 15:e12830. 10.1111/mcn.12830 30989801 PMC6860073

[11] Tekile AK Woya AA Basha GW. Prevalence of malnutrition and associated factors among under-five children in Ethiopia: evidence from the 2016 Ethiopia Demographic and Health Survey. BMC Res Notes. (2019) 12:391. 10.1186/s13104-019-4444-4 31296269 PMC6624874

[12] Akombi BJ Agho KE Hall JJ Merom D Astell-Burt T Renzaho AMM. Stunting and severe stunting among children under-5 years in Nigeria: a multilevel analysis. BMC Pediatr. (2017):1–16. 10.1186/s12887-016-0770-z PMC5237247 28086835

[13] Tariku A Biks GA Derso T Wassie M Abebe SM. Stunting and its determinant factors among children aged 6–59 months in Ethiopia. Ital J Pediatr. (2017):1–9. 10.1186/s13052-017-0433-1 PMC5735819 29258578

[14] Wenling H Huanqing H Wei Z Aiqun H Qi Y Jiang S. Current status of antenatal care of pregnant women-−8 provinces in China, 2018. BMC Public Health. (2021) 21:1135. 10.1186/s12889-021-11154-4 34120600 PMC8201670

[15] Wang L Gao B Hu Y Huang W Cui S. Environmental effects of sustainability-oriented diet transition in China. Resour Conserv Recycl. (2020) 158:104802. 10.1016/j.resconrec.2020.104802

[16] Chen M He W Fu Z Wang Y. Multiple factors analysis on malnutrition of children and under five in different patterns in China in 2000. J Hyg Res. (2003) 32:249–51 10.3969/j.issn.1000-8020.2003.03.018 (in Chinese). 12914291

[17] Liu H Fang H Zhao Z. Urban-rural disparities of child health and nutritional status in China from 1989 to 2006. Econ Hum Biol. (2013) 11:294–309. 10.1016/j.ehb.2012.04.010 22608863 PMC4104502

[18] Department Department of Maternal Child Community Health Ministry Ministry of Health. Reference Standard for Growth and Development of Children Under 7 Years Old in China. (2009). Available online at: http://www.moh.gov.cn-2009-06-02 (in Chinese) (accessed March 15, 2021).

[19] Chen LM Liu GL. Investigation on height development and related factors of 2 529 children aged 6–11 years in Hangu district of Tianjin. J Tianjin Med Univ. (2015) 21:258–61 (in Chinese).

[20] Liu MH Kuang XY Gong Jun Li WJ Zhong X Xie YY. Relationship between family parenting and physical growth and development of children and its influencing factor. J Nanchang Univ. (2014) 54:79–84. 10.13764/j.cnki.ncdm.2014.10.024 (in Chinese)

[21] Jiang Y Su X Wang C Zhang L Zhang X Wang L. Prevalence and risk factors for stunting and severe stunting among children under three years old in mid-western rural areas of China. Child Care Health Dev. (2014) 41: 45–51. 10.1111/cch.12148 24797895

[22] Fetal Medicine Subgroup Society Society of Perinatal Medicine Chinese Medical Association; Obstetrics Subgroup Society Society of Obstetrics and Gynecology Chinese Medical Association. Expert consensus on fetal growth restriction. Chin J Perinat Med. (2019) 22:361–80. 10.3760/cma.j.issn.1007-9408.2019.06.001 (in Chinese).

[23] Aryastami NK Shankar A Kusumawardani N Besral B Jahari B Achadi. Low birth weight was the most dominant predictor associated with stunting among children aged 12–23 months in Indonesia. BMC Nutr. (2017) 3:16. 10.1186/s40795-017-0130-x

[24] Christian P Lee SE Angel MD Adair LS Arifeen SE Ashorn P. Risk of childhood undernutrition related to small-for-gestational age and preterm birth in low- and middle-income countries. Int J Epideiol. (2013) 42:1340–55. 10.1093/ije/dyt109 23920141 PMC3816349

[25] Black RE Victora CG Walker SP Bhutta ZA Christian P Onis M. Maternal and child undernutrition and overweight in low-income and middle-income countries. Lancet. (2013) 382:427–51. 10.1016/S0140-6736(13)60937-X 23746772

[26] Imdad A Yakoob MY Siddiqui S Bhutta ZA. Screening and triage of intrauterine growth restriction (IUGR) in general population and high risk pregnancies: a systematic review with a focus on reduction of IUGR related stillbirths. BMC Public Healtsh. (2011) 11 (Suppl. 11):S1. 10.1186/1471-2458-11-S3-S1 21501426 PMC3231882

[27] Bhutta ZA Das JK Rizvi A Gaffey MF Walker N Horton S. Evidence-based interventions for improvement of maternal and child nutrition: what can be done and at what cost? Lancet. (2013) 382:452–77. 10.1016/S0140-6736(13)60996-4 23746776

[28] Rasmussen KM Stoltzfus RJ. New evidence that iron supplementation during pregnancy improves birth weight: new scientific questions. Am J Clin Nutr. (2003) 78:673–74. 10.1093/ajcn/78.4.673 14522723

[29] Imdad A Bhutta ZA. Routine iron/folate supplementation during pregnancy: effect on maternal anaemia and birth outcomes. Pediatr Perinat Epidemiol. (2012) 26:168–77. 10.1111/j.1365-3016.2012.01312.x 22742609

[30] West KP Christian P Labrique AB Rashid M Shamim AA Klemm RD. Effects of vitamin A or beta carotene supplementation on pregnancy-related mortality and infant mortality in rural Bangladesh: a cluster randomized trial. JAMA. (2011) 305:1986–95. 10.1001/jama.2011.656 21586714

[31] Salam RA Das JK Ali A Lassi ZS Bhutta Z. Maternal undernutrition and intrauterine growth restriction. Expert Rev Obstet Gynecol. (2013) 8:559–67.12098661

[32] Bakketeig L. Current growth standards, definitions, diagnosis and classification of fetal growth retardation. Eur J Clin Nutr. (1998) 52(Suppl. 52): S1–S4 9511013

[33] Geer LA Pycke BF Sherer DM Abulafia O Halden R. Use of amniotic fluid for determining pregnancies at risk of preterm birth and for studying diseases of potential environmental etiology. Environ Res. (2015) 136:470–81. 10.1016/j.envres.2014.09.031 25460669 PMC4279852

[34] Lu G Lin Z Huang J. Association of amniotic fluid contamination, intrauterine fetal distress and neonatal hypoxic-ischemic encephalopathy. China Clin Proct Med. (2019) 10:25–28. 10.3760/cma.j.issn.1673-8799.2019.05.007 (in Chinese)

[35] Ban L Guo S Scherpbier RW Wang XL Zhou H Tata LJ. Child feeding and stunting prevalence in left-behind children: a descriptive analysis of data from a central and western Chinese population. Int J Public Health. (2017) 62:143–51. 10.1007/s00038-016-0844-6 27318527 PMC5288445

[36] Wang A Scherpbier RW Huang X. The dietary diversity and stunting prevalence in minority children under 3 years old: a cross-sectional study in forty-two counties of Western China. BJN. (2017) 118:840–8. 10.1017/S0007114517002720 29189194

[37] Bukusuba J Kaaya AN Atukwase A. Modelling the impact of stunting on child survival in a rural Ugandan setting. BMC Nutr. (2018) 4:1–10. 10.1186/s40795-018-0220-4 32153877 PMC7050845

[38] Irarrázaval B Barja S Bustos E Doirsaint R Senethmm G Guzmn M. Influence of feeding practices on malnutrition in haitian infants and young children. Nutrients. (2018) 10:382. 10.3390/nu10030382 29558413 PMC5872800

[39] Harding KL Aguayo VM Webb P. Birthweight and feeding practices are associated with child growth outcomes in South Asia. Matern Child Nutr. (2018) 14 (Suppl. S4):e12650. 10.1111/mcn.12650 30499248 PMC6972829

[40] Zhang JX Shi L Chen DF Wang J. Effectiveness of an educational intervention to improve child feeding practices and growth in rural China: updated results at 18 months of age. Matern Child Nutr. (2013) 9:118–29. 10.1111/j.1740-8709.2012.00447.x 23020102 PMC6860631

[41] Hart N. Maternal nutrition and infant mortality: a re-examination of the Dutch hunger winter. Popul Stud. (1993) 47:27–46. 10.1080/0032472031000146716 11623195

[42] Liang XH Qi XB Cheng L Zhao HZ. Influencing factors of growth retardation in children under 3 years old in Zhuhai area. Matern Child Health Care China. (2014) 16:2541–4. 10.7620/zgfybj.j.issn.1001-4411.2014.16.29 (in Chinese)

[43] Boah M Azupogo F Amporfro DA Abada LA. The epidemiology of undernutrition and its determinants in children under five years in Ghana. PLoS ONE. (2019) 14:e0219665. 10.1371/journal.pone.0219665 31365528 PMC6668784

