# Supplementary material for: The Effect of Risk Accumulation on Childhood Stunting: A Matched Case-Control Study in China
Source: Front Pediatr. 2022 May 31;10:816870. doi: 10.3389/fped.2022.816870 (PMC9194815; doi:10.3389/fped.2022.816870)
Supplement: Supplementary file 1 [file Table_1.DOCX]

Supplementary Table 1 The independent factors for stunting in the conditional logistic regression

| Independent factors | P | OR | 95% CI |
| --- | --- | --- | --- |
| [Paternal](javascript:;) bearing age^a^ | 0.012 | 2.682 | 1.244,5.778 |
| Maternal height^b^ | 0.001 | 14.986 | 3.216, 69.820 |
| Birth length^c^ | <0.001 | 7.480 | 3.089,18.114 |
| Children's appetite^d^ | 0.010 | 2.154 | 1.252, 5.052 |

^a^ [Paternal](javascript:;) bearing age was ≥35 years old.

^b^Maternal heigh was short vs. not short.

^c^Birth length was short vs. not short.

^d^Children's appetite was good vs. bad/common.
